# A new CUT&RUN low volume-urea (LoV-U) protocol optimized for transcriptional co-factors uncovers Wnt/β-catenin tissue-specific genomic targets

**DOI:** 10.1242/dev.201124

**Published:** 2022-11-30

**Authors:** Gianluca Zambanini, Anna Nordin, Mattias Jonasson, Pierfrancesco Pagella, Claudio Cantù

**Affiliations:** ^1^ Wallenberg Centre for Molecular Medicine, Linköping University, Linköping SE-58183, Sweden; ^2^ Department of Biomedical and Clinical Sciences (BKV), Division of Molecular Medicine and Virology (MMV), Faculty of Medicine and Health Sciences, Linköping University, Linköping SE-58183, Sweden

**Keywords:** CUT&RUN, Chromatin, Transcription, Transcriptional regulation, WNT signaling, β-Catenin

## Abstract

Upon WNT/β-catenin pathway activation, stabilized β-catenin travels to the nucleus where it associates with the TCF/LEF transcription factors, constitutively bound to genomic Wnt-responsive elements (WREs), to activate target gene transcription. Discovering the binding profile of β-catenin is therefore required to unambiguously assign direct targets of WNT signaling. Cleavage under targets and release using nuclease (CUT&RUN) has emerged as prime technique for mapping the binding profile of DNA-interacting proteins. Here, we present a modified version of CUT&RUN, named LoV-U (low volume and urea), that enables the robust and reproducible generation of β-catenin binding profiles, uncovering direct WNT/β-catenin target genes in human cells, as well as in cells isolated from developing mouse tissues. CUT&RUN-LoV-U outperforms original CUT&RUN when targeting co-factors that do not bind the DNA, can profile all classes of chromatin regulators and is well suited for simultaneous processing of several samples. We believe that the application of our protocol will allow the detection of the complex system of tissue-specific WNT/β-catenin target genes, together with other non-DNA-binding transcriptional regulators that act downstream of ontogenetically fundamental signaling cascades.

## Teaser

**Summary:** Gene regulation is governed by protein complexes composed of DNA-binding transcription factors and non-DNA-binding co-factors. CUT&RUN-LoV-U facilitates the detection of the genome-wide binding profile of co-factors.

## INTRODUCTION

Gene regulation is achieved by a combinatorial system of DNA-binding transcription factors (TFs), which physically associate with specific DNA sequences within regulatory regions in the genome, and non-DNA-binding co-factors (co-Fs), which are recruited by TFs or histone marks and serve as hubs to tether chromatin-modifying complexes and RNA polymerase II (Klemm et al., 2019). The molecular apparatus necessary for gene transcription is therefore constituted by a complex assembly of several proteins that recruit each other to the DNA, while not necessarily binding to it (Mitsis et al., 2020; Nikolov and Burley, 1997). Characterizing the genome-wide binding profile and positioning of all these classes of chromatin interactors is crucial to understanding the complexities of gene regulation and its dynamics (Merika and Thanos, 2001).

This is emphasized in the WNT/β-catenin signaling pathway: an evolutionarily conserved intracellular cascade where the extracellular signal carried by WNT ligands is transduced in the signal-receiving cell into a gene expression program (Rim et al., 2022). Here, β-catenin serves as the pivotal protein for signal transduction from the cytosol into the nucleus, where it coordinates a transcriptional response (Valenta et al., 2012). By its nature and structure, β-catenin mediates protein-protein interactions, but it is not capable of directly binding to DNA (Huber et al., 1997). The locus-specific interaction of β-catenin is conferred by its physical association with the TCF/LEF family of TFs, which bind a consensus sequence found within WNT-responsive elements (WREs) (Mosimann et al., 2009). Subsequently, β-catenin recruits a series of co-Fs, such as BCL9/BCL9L, PYGO1/2 (Kramps et al., 2002) and CBP/p300 (Takemaru and Moon, 2000), to engage the basic machinery of transcription and activate target genes (Söderholm and Cantù, 2020). As a consequence, chromatin-associated β-catenin becomes embedded within a large transcriptional complex, sometimes referred to as the WNT enhanceosome (van Tienen et al., 2017). The detection of the chromatin-binding profile of β-catenin has historically been challenging. When using chromatin immunoprecipitation followed by sequencing (ChIP-seq; Furey, 2012), the addition of double-crosslinking steps aimed at preserving not only the DNA-protein but also the protein-protein interactions, yielded clear β-catenin profiles both *in vitro* (Schuijers et al., 2014) and *in vivo* (Cantù et al., 2018). These protocols, however, present several limitations, including the fact that they are at risk of generating numerous artifacts and, most importantly, require enormous amounts of cells – typically in the range of tens of millions (Doumpas et al., 2019; Zimmerli et al., 2020).

Among the several technologies recently developed to profile DNA-binding proteins, cleavage under targets and release using nuclease (CUT&RUN, hereafter C&R) has emerged as method of choice, as it does not require cross-linking and yields genome-wide TF profiling from a significantly lower cell input than ChIP-seq (Skene and Henikoff, 2017; Skene et al., 2018). C&R relies on the antibody-mediated recognition of specific target factor by the fusion of proteinA/G, a recombinant protein combining the affinity for the heavy antibody chains of both protein A and protein G, with micrococcal nuclease (pAG-MN), which cuts the DNA in a sequence-independent manner upon addition of Ca^2+^. When activated, pAG-MN therefore cleaves the DNA underlying target TFs and generates short fragments that diffuse into the supernatant, which can be harvested and sequenced, before mapping onto a reference genome – producing TF-specific genome-wide binding patterns (Meers et al., 2019a).

In our attempts to characterize the genomic positioning of the WNT/β-catenin regulators, C&R systematically failed in producing a binding profile of the non-DNA-binder β-catenin. To solve this limitation, we tested a number of biochemical adaptations in select steps of the C&R protocol to finally generate a modified procedure referred to as C&R-LoV-U (low volume and urea). C&R-LoV-U uses nuclear extraction and *in situ* protein denaturation to improve retrieval of DNA fragments that are associated with transcriptional complexes. Reduced volumes and a streamlined pipeline further confer high reproducibility and scalability to the protocol. Importantly, C&R-LoV-U not only allows detection and profiling of β-catenin, but also improves DNA fragment retrieval when other non-DNA-binding co-Fs are targeted, and it is transferrable to all the other classes of chromatin binding proteins tested, such as histone modifications, classical TFs and other components of large multi-protein complexes. We employed C&R-LoV-U on cultured HEK293T cells, as well as *ex vivo* in cells isolated from mouse developing hindlimbs, which provide a relatively small subset of cells with active WNT signaling (Maretto et al., 2003). We propose that C&R-LoV-U will permit the study of a broad spectrum of chromatin regulators that differ based on DNA-binding affinity, proximity to DNA, and position within large multi-protein complexes. Moreover, we envision that systematic application of this protocol will allow the study of the full set of non-DNA-binding transcriptional regulators that act downstream of ontogenetically fundamental signaling cascades.

## RESULTS AND DISCUSSION

### *In situ* urea administration on isolated nuclei allows the release of β-catenin-associated DNA fragments

**Fig. 1.**
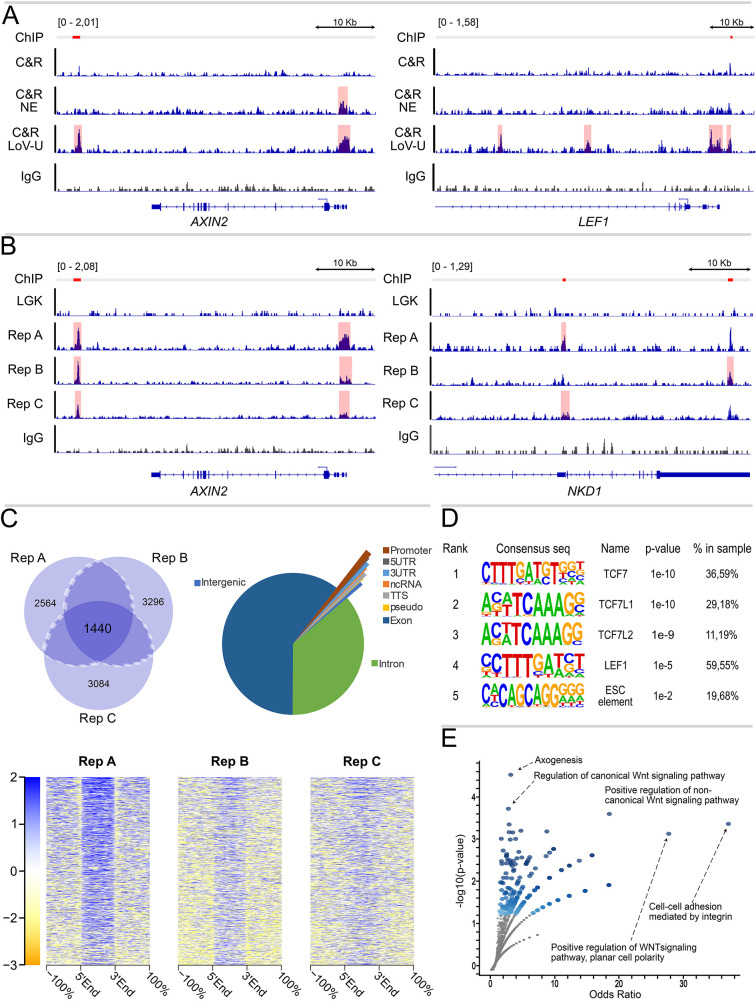
**CUT&RUN with nuclear extraction and urea-mediated release allows reproducible profiling of β-catenin binding in HEK293T.** (A) Genome coverage tracks of two different loci of traditional CUT&RUN (C&R) for β-catenin, C&R with nuclear extraction (C&R NE) and C&R using urea in the elution buffer (C&R-LoV-U: low volume-urea, see Fig. 2 for a complete explanation of the protocol) for β-catenin and IgG-negative control, scaled to signal per million reads. Peak regions called by SEACR are shaded. C&R-LoV-U shows enriched signal compared with traditional C&R and C&R NE for β-catenin, and successfully recapitulates previously published ChIP-seq peaks at known WNT-responsive elements, indicated by red lines corresponding to the exact positions of the β-catenin peaks called in Doumpas et al. (2019). (B) Genome coverage tracks of three biological replicates (Rep A-C) of the C&R-LoV-U β-catenin and IgG-negative control, showing reproducible signal enrichment across replicates in two different loci. These peaks are not recapitulated in data from C&R LoV-U β-catenin under LGK conditions (Pagella et al., 2022 preprint), when WNT is not active. (C) Left: Venn diagram of number of peaks called by SEACR for β-catenin replicates. The peaks called in two out of three replicates were considered reproducible. Reproducible peaks under LGK conditions were subtracted from these peaks, and the subsequent peak set was considered high-confidence and used for downstream analyses. Right: pie chart showing genomic region annotations for β-catenin peaks. Bottom: signal enrichment plots displaying fold-change over IgG control for each replicate over the high-confidence peak regions. Signal entries in the heatmap are ordered by overall enrichment of the first profile. (D) Motif analysis results for high-confidence β-catenin peak regions, showing significant enrichment for TCF/LEF-binding motifs. (E) Gene ontology analysis of the peak-associated genes, where the odds ratio (ratio of input list to reference list, *x*-axis) and the statistical significance (*y*-axis) for groups of ‘GO-biological processes’ are represented, shows enrichment for several WNT pathway-related mechanisms.

A reliable method to trigger Wnt/β-catenin activation is treatment with CHIR99021 (hereafter referred to as CHIR), a potent GSK3 inhibitor that causes β-catenin stabilization. CHIR stimulation in HEK293T cells generates a reproducible genome-wide DNA-binding profile of β-catenin, detectable via ChIP-seq (Doumpas et al., 2019). In our hands, C&R repeatedly failed to recapitulate the β-catenin ChIP-seq profile in this cell line, generating, at best, sub-optimal enrichment regions that do not allow consistent peak calling (Fig. 1A). We reasoned that β-catenin might constitute a difficult C&R target mainly for three reasons. First, when WNT signaling is active, β-catenin must build up in the cytosol before being translocated to the nucleus. We suspect that the high level of cytosolic β-catenin could sequester the added antibody and subsequent pAG-MN, hindering its ability to reach nuclear β-catenin and to cleave DNA. The second reason could be its physical distance from DNA, which might not allow pAG-MN to simultaneously reach its target (i.e. the antibody against β-catenin) while cleaving the underlying genomic region. A third explanation might reside in the positioning of β-catenin within a multi-protein complex, which would hinder or retard the release into solution of any cleaved DNA fragments. We consider the second explanation unlikely, as the use of a secondary antibody, as previously employed to extend the reach of pAG-MN (Hainer and Fazzio, 2019), did not improve the experimental outcome when tested. On the other hand, while nuclei isolation alone only marginally improved the final yield (Fig. 1A), it was the subsequent implementation of chaotropic agents – molecules that cause protein denaturation – that, when applied after pAG-MN cleavage, enabled the release and harvest of a considerably higher amount of DNA fragments that correctly mapped to WREs in the vicinity of WNT target genes (Fig. 1A). Based on this finding, we developed an elution buffer in which a high final concentration of urea (up to 8.8 M) proved optimal in maximizing the recovery of DNA fragments associated with the pAG-MN-dependent cleavage pattern produced when specifically targeting β-catenin (Fig. 1A, Table S1). Key comparisons support the reliability of our protocol modification. First is the recapitulation of classical β-catenin-binding targets, as observed in previous ChIP-seq assays (Fig. 1B; Doumpas et al., 2019). Second is the reproducibility of the protocol, providing comparable signal tracks (Fig. 1B) and peak enrichment across three replicates (Fig. 1C) in primarily intergenic and intronic regions, as found previously (Fig. 1C; Hoverter et al., 2014; Cantù et al., 2018). Third is the statistical enrichment for TCF/LEF motifs as primary transcription factor signature in the sequence underlying the high confidence β-catenin peaks (called in at least two out of three replicates as described by Yang et al., 2014; Fig. 1D). Last is the prevalence of WNT pathway-related Gene Ontology categories across peak associated gene sets (Fig. 1E). It is relevant noting that, in the original C&R protocol (Skene and Henikoff, 2017), the authors successfully purified chromatin-associated protein complexes. To achieve this, they found it necessary to extract total DNA, rather than only the solubilized fragments, to then remove large DNA fragments with size-selection beads-based reagents. We believe that *in situ* protein denaturation via urea exerts an analogous effect without the need for whole-genome purification and the subsequent costly bead-mediated short-fragment enrichment.

**Fig. 2.**
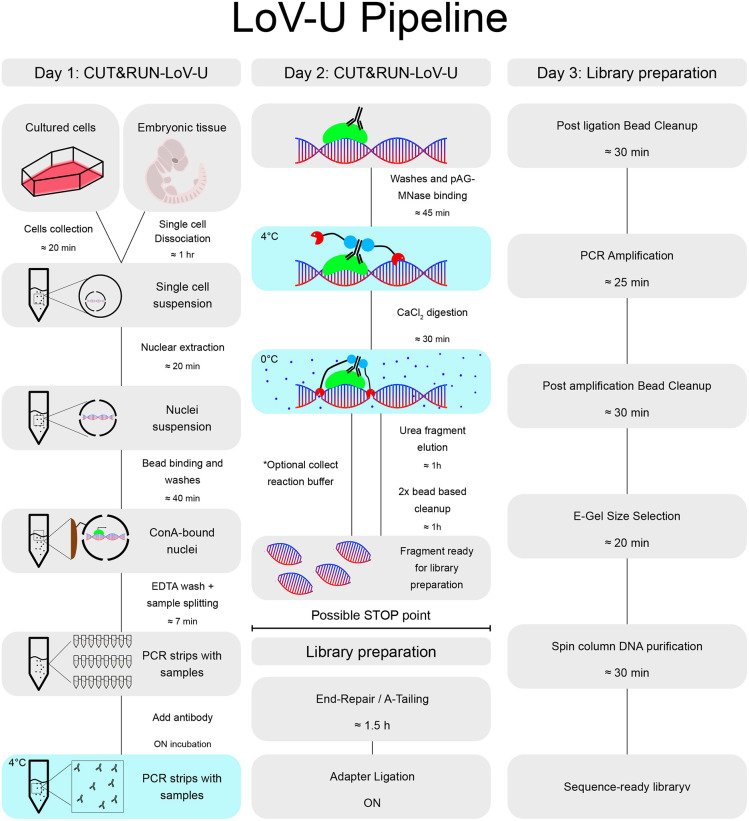
**Workflow for the CUT&RUN low volume and urea (C&R LoV-U) protocol.** Day 1: cells are harvested from adherent culture or embryonic tissue and dissociated to a single cell suspension. Nuclei are extracted and bound to concanavalin A (ConA) beads, and the sample is split into PCR tubes for each condition. Antibodies are added and samples are incubated overnight. Day 2: all steps are performed in PCR tubes with a multi-channel pipette. First, excess antibodies are washed away and the samples are incubated with pAG-MNase. Excess pAG-MNase is removed, then calcium is added to induce pAG-MNase digestion, which proceeds for 30 min. A buffer containing urea is added as a chaotropic agent to the nuclei preparation, allowing *in situ* protein solubilization and release of protein-bound chromatin fragments. For factors with smaller expected fragment profiles, the digestion buffer can be retained and added back before purification. Bead-based DNA purification removes contaminants and renders samples suitable for library preparation. End-repair and A-tailing are performed, followed by overnight adapter ligation. Day 3: library preparation continues in PCR tubes. Post-ligation bead clean-up, library amplification, post-amplification bead clean-up, gel-based size selection and DNA purification can be performed in 2-3 h, resulting in sequencing-ready DNA libraries. With the gel-based size selection, any adapter-dimer contamination can be excluded and DNA fragment size estimated.

### Reduced volumes and design of 3D-printable magnetic racks improve scalability – the low volume and urea (LoV-U) protocol

During the optimization of the protocol, we strived to include additional modifications that would allow us to simultaneously streamline the procedure, and improve scalability and throughput of the technique, thereby permitting the concurrent profiling of a higher number of samples (Fig. 2). These modifications were mostly designed to decrease reaction volumes; for example, reducing wash volume but increasing number of washing steps yielded similar signal-to-noise ratios, but permitted us to perform the entire protocol using 200 µl PCR tubes and multichannel pipettes. The low volumes (LoV) parallel processing improved the procedure speed and its cost-effectiveness (i.e. it is possible to reduce the amount of reagents used proportionally to the final volume), and it promoted reproducibility across replicates (Fig. 2). To facilitate washes and buffer changes, C&R uses magnetic beads as a substrate and thus requires the use of a magnetic tube rack. Accordingly, we designed and 3D printed magnetic racks that provide a better fit for the processing of eight samples simultaneously at virtually no additional cost. As the use of these is an integral part of our procedure, we have included the design of magnetic racks and all the necessary instructions for their 3D printing in supplementary File S1.

### C&R-LoV-U enables profiling across different types of protein targets

**Fig. 3.**
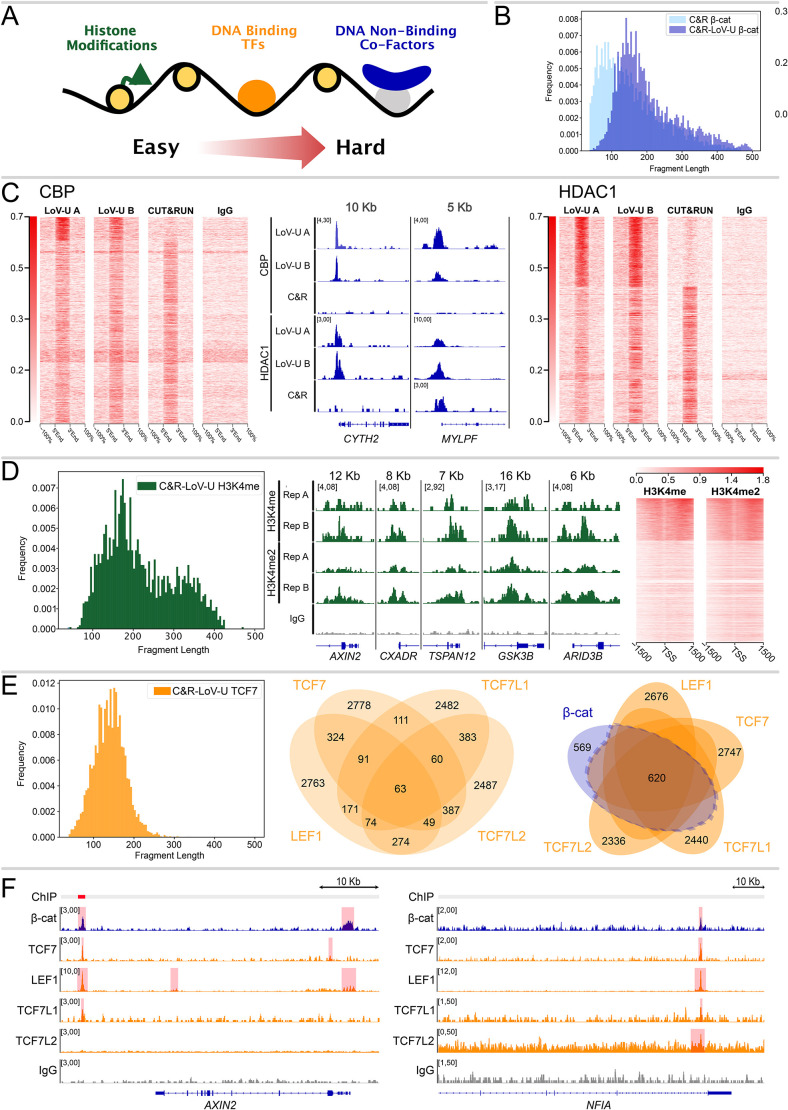
**C&R-LoV-U is well adapted to the entire spectrum of chromatin-associated targets.** (A) Schematic representation of the proposed difficulty for targets of chromatin-profiling techniques. (B) Histogram showing fragment length distribution for traditional C&R and C&R-LoV-U against β-catenin; C&R-LoV-U results in a comparatively larger fragment size. (C) Left: signal enrichment plots for CBP carried out with the C&R-LoV-U and traditional C&R protocols, showing combined peaks from all replicates. C&R-LoV-U recapitulated all signal enrichment from C&R and additionally contained enriched regions not found in C&R. Signal entries in the heatmap are ordered by overall enrichment of the first profile. Middle: Example of a locus containing peaks in CBP and HDAC1 that were present in only LoV-U (left) and a locus where a peak is also present in classical C&R for HDAC1 but not CBP (right). The overlap between the LoV-U replicates is much higher than that for the C&R. Right: signal enrichment plots for HDAC1 carried out with the C&R-LoV-U and traditional C&R protocols, showing combined peaks from all replicates. The strongest peaks, replicated in both C&R-LoV-U datasets, were absent in the C&R data. (D) Left: histogram of fragment length for C&R LoV-U against H3K4me2, showing both nucleosomal (∼150 bp) and di-nucleosomal (∼300 bp) fragments. Middle: genome coverage tracks scaled to signal per million reads showing two replicates of C&R-LoV-U for H3K4me and H3K4me2 near the transcriptional start site (TSS) of several loci. Right: signal enrichment plots for H3K4me and H3K4me2 around the TSS for all Refseq genes. Signal entries in the heatmap are ordered by overall enrichment of the first profile. (E) Left: histogram of fragment length for C&R-LoV-U against TCF7, showing both subnucleosomal (<120) and nucleosomal fragments. Middle: Venn diagram of overlap of SEACR called peaks for the TCF/LEF transcription factors (*n*=1). Right: Venn diagram of the overlap between high-confidence β-catenin peaks and the TCF/LEFs. Over half of β-catenin peaks are called in at least one TCF/LEF dataset. (F) Genome coverage tracks scaled to signal per million reads showing C&R-LoV-U against β-catenin, TCF7, LEF1, TCF7L1, TCF7L2 and IgG-negative control. At the *Axin2* enhancer, peaks are called in three out of four TCF/LEFs and some signal enrichment can be seen in all tracks, while the promoter of *Nfia* contains called peaks for β-catenin and all four TCF/LEF factors.

We hypothesize that different classes of protein targets present different challenges to detection using C&R and similar techniques (Fig. 3A). For example, chromatin marks and histone modifications are successfully detected with an extremely low number of cells, likely due to their abundance and the breadth of the chromatin region exposing their epitope (Calo and Wysocka, 2013; Meers et al., 2019a). TFs, conversely, are less abundantly expressed, are located at precise positions on the chromatin and are more challenging to detect (Furey, 2012). Finally, non-DNA binders would constitute the most difficult type of target, as exemplified by our C&R attempts and the previous need for ChIP-seq protocols to adopt a dual cross-linking approach (Cantù et al., 2018; Salazar et al., 2019; Schuijers et al., 2014). Consistently, to our knowledge only a few C&R attempts targeting co-Fs have been reported (Beon et al., 2022). Therefore, we tested whether C&R-LoV-U could adequately detect other non-DNA-binding transcriptional co-Fs, in addition to β-catenin. We selected the histone acetyl transferase CBP (also known as CREB-binding protein) and the deacetylase HDAC1, as they were among the first functionally relevant proteins tethered to the WRE by association with β-catenin (Billin et al., 2000; Hecht et al., 2000; Olson et al., 2006; Takemaru and Moon, 2000). Classical C&R was capable of detecting a reliable binding profile of both these non-DNA binding proteins (Fig. 3C). C&R-LoV-U, on the other hand, could not only recapitulate the tracks obtained by original C&R results, but also revealed an additional set of peaks (Fig. 3C). Notably, this new fraction of peaks represents the group with the highest local signal-to-noise ratio (Fig. 3C). This suggests that C&R-LoV-U facilitates the extraction of DNA fragments from chromatin regions that are accessible to pAG-MN but, likely due to local crowding, do not allow release of the cut DNA. We speculate that this might underlie the differential recruitment of co-Fs at genomic loci that have different biophysical properties and protein concentrations, and that this influences their detectability. C&R-LoV-U, by *in situ* protein denaturation, might ‘break’ these phase-separated clusters and permit diffusion of water-soluble molecules.

The results presented above suggest that our protocol could also perform well when applied to ‘easier’ targets than non-DNA-binding co-Fs. However, we noticed that, when targeting β-catenin, C&R-LoV-U resulted in an enrichment of supra-nucleosomal DNA fragments (>150 nucleotides), larger than those obtained with the original C&R targeting β-catenin (Fig. 3B). Classical TFs, on the other hand, typically release smaller, sub-nucleosomal, DNA fragments (Meers et al., 2019b). Therefore, we aimed to test whether C&R-LoV-U could be employed for other types of targets in addition to co-Fs. As shown in Fig. 3, our protocol successfully profiled the other two classes of targets: (1) histone post-translational modifications (H3K4me and H3K4me2); and (2) DNA-binding TFs (the WNT signaling relevant LEF1, TCF7, TCF7L1 and TCF7L2). C&R LoV-U for H3K4me and H3K4me2 yielded the expected fragment lengths corresponding to nucleosomal and di-nucleosomal sizes, as described by Meers et al. (2019b), together with widespread enrichment downstream of transcriptional start sites, as described by Soares et al. (2017) (Fig. 3D). When targeting TFs, the length distribution of the obtained fragments was consistent with average smaller size and included both sub-nucleosomal and nucleosomal fragments (Fig. 3E). Moreover, when the reads were mapped to a reference genome, the binding profiles for the different TCF/LEF transcription factors partially overlapped with each other (Fig. 3E,F), consistent with their known partial redundancy (Cadigan and Waterman, 2012; Moreira et al., 2017). Additionally, considerable overlap between the high confidence β-catenin peaks and the TCF/LEF peaks lends credibility to these datasets, while the presence of β-catenin peaks that are seemingly independent of TCF/LEF could be due to peak calling discrepancies, but also aligns with previous findings (Doumpas et al., 2019) (Fig. 3E). Unique peaks for the different TCF/LEF TFs are likely due to a combination of locus specificity and imperfect peak calling (Fig. 3E). As shown above, our improved C&R-LoV-U protocol consistently performs on all the types of targets tested and is therefore suitable for genome-wide profiling of all chromatin-interacting proteins.

### β-Catenin profiling *in vivo* in developing mouse tissue uncovers tissue-specific targets

**Fig. 4.**
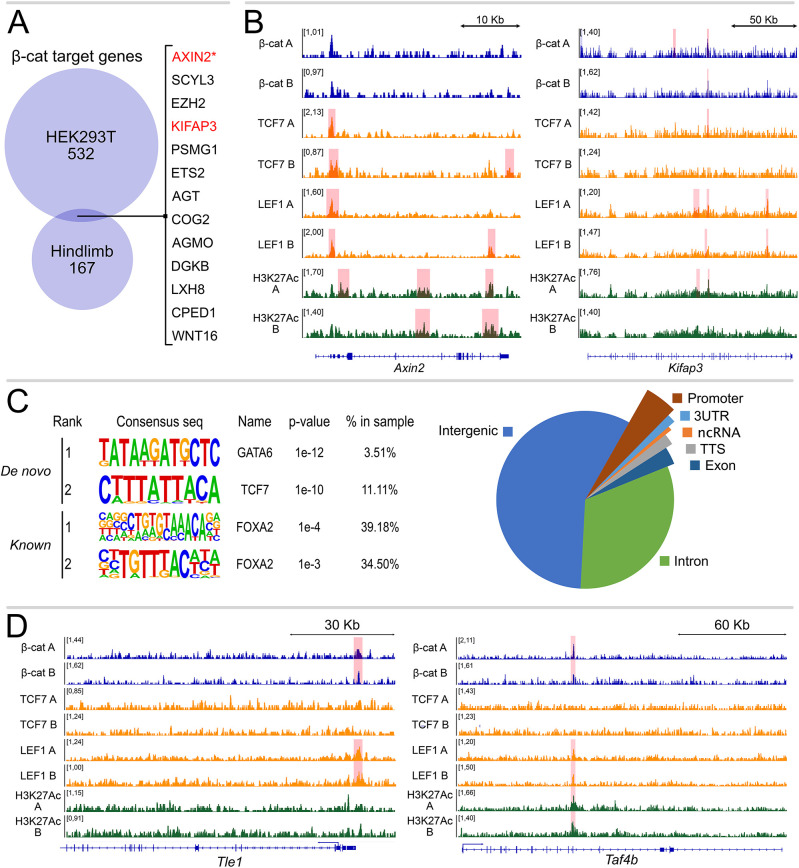
**C&R-LoV-U uncovers a hindlimb-specific β-catenin-binding profile.** (A) Overlap of GREAT annotated target genes for HEK293T β-catenin peaks and hindlimb at 11.5 days post coitum (dpc) β-catenin peaks. Only 12 genes are shared, demonstrating high tissue-specificity of β-catenin activity. Genes in red are shown in B and the asterisk indicates that Axin2 is not called as a peak by SEACR. (B) C&R-LoV-U genome coverage tracks for β-catenin, TCF7, LEF1 and H3K27ac in hindlimb at the *Axin2* and *Kifap3* loci, scaled to signal per million reads. Signal enrichment can be seen at the *Axin2* promoter and intronic enhancer regions of *Kifap3*. (C) Left: motif analysis of hindlimb β-catenin peaks, showing enrichment of GATA6 and FOXA factors in addition to TCF/LEF. Right: pie chart with peak region annotations of hindlimb β-catenin peaks. (D) C&R-LoV-U genome coverage tracks for β-catenin, TCF7, LEF1 and H3K27ac in hindlimb at the *Tle1* and *Taf4b* loci, scaled to signal per million reads. Signal enrichment for β-catenin and LEF1 can be seen at the promoter of *Tle1*, while enrichment of β-catenin, LEF1 and H3K27ac can be seen within an intronic region of *Taf4b*, which is likely an enhancer.

We aimed at testing the suitability of our protocol in an ontogenically relevant tissue extracted during mouse organogenesis. We selected developing hindlimbs at 11.5 days post coitum (dpc), because β-catenin is required to initiate the gene expression program towards the formation of this organ (Kawakami et al., 2011) and because hindlimbs display a relatively thin layer of cells with active WNT signaling (Maretto et al., 2003). By employing C&R-Lov-U, we successfully identified 171 β-catenin peaks, present in two biological replicates, which were annotated by GREAT (McLean et al., 2010) to 179 genes (Fig. 4, Table S1). To our surprise, of these, only 12 were in common with the annotated target genes identified in HEK293T cells, while 167 appeared to be specific to the hindlimb (Fig. 4A). We noticed that the stringency of our peak calling might cause a reduced overlap between the HEK293T and the hindlimb peaks, thereby leading to overestimation of the tissue-specific subsets. For example, by using the same statistical parameters, *Axin2* was not called as a gene-associate peak in the hindlimb, despite the consistent signal enrichment observed at its promoter (Fig. 4B). This is possibly caused by the intrinsically lower signal-to-noise ratio obtained when targeting β-catenin in mouse hindlimb cells, likely reflecting the higher heterogeneity of this cell population in comparison with HEK293T, and the small proportion of cells with physiologically active WNT/β-catenin signaling (Maretto et al., 2003). Therefore, we manually searched the list for hindlimb-specific genes to examine whether previously neglected β-catenin targets might include some that are known to be relevant for formation of this organ. Interestingly, we found notable targets such as *Ets2* and *Ezh2* (Fig. 4A), both of which have been shown to be involved in mouse limb development (Ristevski et al., 2002; Wyngaarden et al., 2011). Another novel target of interest is *Kifap3* (Fig. 4B)*.* Here, the β-catenin peak does not correspond to its exact location in HEK293T (by comparing the mouse and the human homologue genomic sequences), but it lies within a different intron of the gene. Of note however, LEF1 also associates with these genomic loci (Fig. 4B), thereby increasing our confidence in the reliability of these binding events even in regions of relatively low signal. Of relevance, the introns of *Kifap3* have been shown to contain a limb-specific enhancer that is activated only during mouse limb formation (Nolte et al., 2014). Our data establish that this limb-relevant ensemble of developmental regulatory regions is likely regulated by canonical WNT/β-catenin signaling. Similar to the HEK293T peak set, the hindlimb binding regions are primarily located in intergenic and intronic regions of the genome (Fig. 4C). We performed motif analysis on the hindlimb peak set and identified, in addition to TCF/LEF motifs, enrichment of the motifs of other TFs, such as GATA6 and FOXA (Fig. 4C). Notably, GATA4 and GATA6 are present in an anterior-posterior gradient in the forelimb, but only GATA6 is expressed in hindlimb buds, where it acts as inhibitor of *Shh* (Kozhemyakina et al., 2014). Our data therefore unearth a potential GATA6-WNT/β-catenin interplay that is crucial to balancing the levels of SHH and ultimately determining digit patterning. We identified several other hindlimb-only target genes that display β-catenin binding in their regulatory regions and manually checked that these regions do not show β-catenin enrichment in HEK (Fig. S1C). Among these were the following: *Tle1*, encoding the transducin-like enhancer of split/groucho repressor and known to bind to TCF/LEF at WREs and to repress transcription of WNT target genes, pointing to a potential novel negative-feedback mechanism (Fig. 4D) (Sekiya and Zaret, 2007); *Igf1*, which is known to play a role in chondrogenesis and apoptosis in the developing limb (Fig. S1B) (van Kleffens et al., 1998); *Taf4b*, which is a basal transcription factor downstream of TGFβ signaling and highly expressed in the limbs during development (Fig. 4D) (Iwata et al., 2010); and *Shox2*, which is a transcription factor required for proper bone and muscle development in the mouse limbs (Fig. S1B) (Vickerman et al., 2011). The identification of tissue-specific physical targets of β-catenin does not imply that these constitute functionally relevant transcribed genes, as previous studies have shown that context-dependent mechanisms are necessary for transcription after β-catenin recruitment (Nakamura and Hoppler, 2017; Nakamura et al., 2016). Taken together, our datasets indicate that C&R-LoV-U possesses the sensitivity to identify both common and tissue-specific β-catenin direct target genes, and that application of this protocol will allow the determination of how a seemingly universal transcriptional cascade can regulate differential sets of target genes depending on the cellular context.

## MATERIALS AND METHODS

### Cell culture

Human embryonic kidney 293T cells (HEK293T) were obtained by previous work (Doumpas et al., 2019). HEK293T were cultured at 37°C in a humidified incubator with 5% CO_2_. Cells were cultured in high glucose Dulbecco's Modified Eagle Medium (41965039, Gibco) supplemented with 10% bovine calf serum (1233C, Sigma-Aldrich) and 1× penicillin-streptomycin (15140148, Gibco).

### Animal experimentation and tissue processing

Animal housing and experimentations were performed according to the Swedish laws and guidelines under the ethical animal work license obtained by C.C. at Jordbruksverket (Dnr 2456-2019). JAX Swiss Outbred mice (strain 034608) were used for all experiments. Animals were kept in Allentown NexGen IVCs, floor area 500 cm^2^. The maximum number of mice was four per cage. Cages were supplied with aspen wood shavings as bedding and two types of shredded paper as nesting material. Paper tubes were provided as additional enrichment. Temperature was set at 21±2°C, humidity to 45-65% and light cycle to 12 h/12 h (7.00 am/7.00 pm). The animals had unrestricted access to sterilized drinking water, and *ad libitum* access to a pelleted and extruded mouse diet in the food hopper. Mice were housed in a barrier-protected specific pathogen-free unit. The specific pathogen-free status of the animals was monitored frequently and confirmed according to FELASA guidelines by a sentinel program. The mice were free of all viral, bacterial and parasitic pathogens listed in FELASA recommendations (Mähler et al., 2014). The age of embryos was determined according to timed mating and vaginal plug observation (0.5 dpc), and confirmed by morphological criteria. Pregnant females were sacrificed by cervical dislocation and E11.5 embryos were surgically removed. Hindlimbs were dissected under a dissection stereomicroscope (SZ61, Olympus), pooled and dissociated to single cells via incubation in TrypLE Express Enzyme (12-604-013, Thermo Fisher Scientific; 1 ml/4 limbs) for 15 min at 37°C on a shaker. The cell suspension was resuspended in ice-cold PBS, filtered through a 40 µm cell strainer (KKE3.1, Carl Roth) and further processed for CUT&RUN.

### CUT&RUN

CUT&RUN was performed as described by Skene et al. (2018). Before harvest, HEK293T cells were cultured in media containing 10 µM CHIR99021 (SML1046, Sigma-Aldrich) for 24 h; cells used for HDAC1 and CBP were incubated for 4 h. 500,000 cells were harvested using TrypLE (12604013, Gibco), washed twice in wash buffer [20 mM HEPES (pH 7.5), 150 mM NaCl, 0.5 mM spermidine in Roche Complete Protease Inhibitor EDTA-Free (COEDTAFRO)] and bound to 20 µl magnetic ConA agarose beads (ABIN6952467, antibodies-online) equilibrated in binding buffer [20 mM HEPES (pH 7.5), 10 mM KCl, 1 mM CaCl_2_ and 1 mM MnCl_2_]. Cells for the β-catenin nuclear extraction CUT&RUN sample were nuclear extracted and bound to beads according to the protocol in CUT&RUN LoV-U, after which the following protocol was used. Antibody incubation was performed in 150 µl antibody buffer (wash buffer with 0.01% digitonin and 2 mM EDTA) with 1.5 µl antibody (see Table S2) overnight at 4°C. Thereafter, washes were performed with wash buffer containing 0.01% digitonin. After overnight incubation, samples were washed three times and pAG-MN protein was added at 0.6 µg/ml, in a total volume of 150 µl. pAG/MNase was a gift from Steven Henikoff (Addgene #123461), expressed and purified as described previously (Meers et al., 2019a). Samples were washed three times, followed by digestion for 30 min in wet ice in wash buffer supplemented with 2 mM CaCl_2_. 2× stop buffer (340 mM NaCl, 20 mM EDTA, 4 mM EGTA, 0.05% digitonin, 100 µg/ml RNase A and 50 µl/ml glycogen) was added to stop the reaction, and samples were incubated for 30 min at 37°C. Beads were collected on the magnet and liquid transferred to a new tube. 2.5 µl proteinase K (P8107S, New England BioLabs) and 2 µl 10% SDS were added, and samples incubated for 1 h at 50°C. 200 µl phenol-chloroform (P3803, Sigma-Aldrich) was added and samples were vortexed before being centrifuged at 15,000 ***g*** for 10 min. The aqueous phase was transferred to a new tube containing 1.5 µl glycogen, 20 µl 3 M sodium acetate and 500 µl 100% ethanol. Samples were incubated overnight at −20°C. DNA precipitation was performed by centrifugation at 15,000 ***g*** for 45 min at 4°C. DNA pellets were washed once in 70% ethanol and centrifuged for 10 min at 15,000 ***g***. Pellets were allowed to air dry before being resuspended in 20 µl Tris-HCl (pH 7.5).

### CUT&RUN LoV-U

The detailed protocol is provided in the supplementary Materials and Methods. HEK293T cells were cultured in media containing 10 µM CHIR99021 for 24 h; cells used for HDAC1 and CBP were incubated for 4 h. Then, 500,000 cells/sample were harvested using TrypLE and washed twice in DPBS (14190094, Thermo Fisher Scientific). For each *ex vivo* sample ∼100,000 cells (three hindlimbs at 11.5 dpc) were collected. Cells were washed three times in nuclear extraction (NE) buffer [20 mM HEPES-KOH (pH 8.2), 10 mM KCl, 0.5 mM spermidine, 0.05% IGEPAL, 20% glycerol, Roche Complete Protease Inhibitor EDTA-Free], resuspended in 40 µl NE per sample and bound to 20 µl magnetic ConA agarose beads equilibrated in binding buffer. After incubation, nuclei and beads were resuspended for 5 min in EDTA wash buffer (wash buffer with 0.2 mM EDTA). Samples were divided into 200 µl PCR tubes and antibody incubation was performed in 200 µl wash buffer with 2 µl of antibody (for antibody and batch information, see Table S2) overnight at 4°C on a rotator. After overnight incubation, samples were washed five times in wash buffer and resuspended in 200 µl of pAG-MN buffer (wash buffer with pAG-MN 0.6 µg/ml) for 30 min at 4°C on a rotator. Samples were washed five times, followed by digestion for 30 min in wet ice in wash buffer with 2 mM CaCl_2_. After 30 min, the digestion buffer was removed and the reaction was stopped with 50 µl of 1× Urea STOP buffer (100 mM NaCl, 2 mM EDTA, 2 mM EGTA, 0.5% IGEPAL, 8.8 M urea) and the samples were incubated 1 h at 4°C. For HDAC1 and CBP, the digestion buffer was saved and later combined with the Urea STOP elution. Beads were collected on the magnet and liquid transferred to a new PCR tube in which it was cleaned up twice using Mag-Bind TotalPure NGS beads (M1327, Omega Bio-Tek) at 2×, and then resuspended in 20 µl Tris-HCl (pH 7.5).

### Library preparation and sequencing

Library preparation was performed using the KAPA Hyper Prep Kit for Illumina platforms (KK8504, KAPA Biosystems) according to the manufacturer's guidelines with the following modifications. End repair and A-tailing was performed in 0.4× volume reactions with 20 µl of purified DNA. The thermocycler conditions were set to 12°C for 15 min, 37°C for 15 min and 58°C for 25 min to prevent thermal degradation of the shortest fragments. Adapter ligation was performed in 0.4× volume reactions. KAPA Dual Indexed adapters were used at 0.15 µM. A post-ligation clean-up was performed with Mag-Bind TotalPure NGS beads at 1.2× the sample volume. Resuspension was carried out in 10 mM Tris-HCl (pH 8.0). Library amplification was performed in 0.5× volume reactions. The cycling conditions were set as follows: 45 s initial denaturation at 98°C, 15 s denaturation at 98°C, 10 s annealing/elongation at 60°C, 1 min final extension at 72°C, hold at 4°C, with 13 cycles. After amplification, a post-amplification clean-up was performed using NGS beads at 1.2× sample volume. Libraries were then run on an E-Gel EX 2% agarose gel (G402022, Invitrogen) for 10 min using the E-Gel Power Snap Electrophoresis System (Invitrogen). Bands of interest between 150 and 500 bp were excised and purified using the GeneJET Gel Extraction Kit (K0691, Thermo Fisher Scientific) according to manufacturer's instructions. Libraries were quantified with the Qubit (Thermo Scientific) using the high sensitivity DNA kit (Q32854, Thermo Fisher Scientific), pooled and sequenced 36 bp pair-end on the NextSeq 550 (Illumina) using the Illumina NextSeq 500/550 High Output Kit v2.5 (75 cycles) (20024906, Illumina) to an approximate depth of 5-10 million reads per sample.

### Data analysis

Quality of reads was assessed using fastqc (Brandine and Smith, 2022, version 0.11.9). Trimming was performed using bbmap bbduk (Bushnell et al., 2017, version 38.18) removing adapters, artifacts, poly AT and TA repeats, and poly G and C repeats. Reads were aligned to the hg38 genome or mm10 genome using bowtie (Langmead et al., 2009, version 1.0.0) with the options -v 0 -m 1 -X 500. Samtools (Li et al., 2009, version 1.11) view, fixmate, markdup and sort were used to create bam files, to mark and remove duplicates, and to sort bam files. Fragment size analysis was carried out using deeptools (Ramírez et al., 2016, version 3.5.1-0) bamPEFragmentsize -hist. Bedgraphs were created using bedtools (Quinlan and Hall, 2010, version 2.23.0) genomecov on pair-end mode. Normalized signal per million reads tracks for visualization were created by removing mitochondrial reads from bam files with awk followed by the use of the --SPRM function of macs2 (Zhang et al., 2008, version 2.2.6) with the options -f BAMPE --keep-dup all --SPMR and –bdg. Peaks were called using SEACR (Meers et al., 2019c, version 1.3) for each bedgraph using the settings norm and stringent with a threshold set to 0.001. Final peak sets were generated by using bedtools subtract with the option -A to remove blacklisted regions (Amemiya et al., 2019) (unified hg38 for hg38, ENCODE v1 for mm10), and regions overlapping the peaks called in the corresponding IgG-negative control for HEK293T samples. Venn diagrams and overlap peak sets were created using Intervene (Khan and Mathelier, 2017, version 0.6.4). CUT&RUN data for β-catenin under LGK conditions, obtained by Pagella et al. (2022 preprint), was downloaded from ArrayExpress (accession number E-MTAB-12077). Mapped reads were downscaled to match average sequencing depth of the β-catenin CHIR samples, after which the samples were processed as described above. High confidence β-catenin peaks were considered those called in at least two out of three replicates in HEK293T in CHIR and LGK. The final high-confidence β-catenin CHIR peaks were considered as only those not reproducible under LGK conditions and were used for further analysis. For comparison with ChIP, high-confidence peak regions were downloaded from Doumpas et al. (2019) and converted from hg19 to hg38 using the UCSC LiftOver (Hinrichs et al., 2006) with default settings. Signal intensity plots for β-catenin were created using ngsplot (Shen et al., 2014, version 2.63) with options -G hg38 -R bed -N 2 -SC global -IN 0 -CD 1 -GO total for each β-catenin replicate against the IgG negative control, for H3K4me and H3K4me2 with the options -G hg38 -R tss -SC global -L 1500 -GO total, and for HDAC1 and CBP with the options -G hg38 -R bed -N 2 -SC global -GO total, using a bed file containing additive peaks from all replicates. Motif analysis was carried out using Homer (Heinz et al., 2010, version 4.11) findMotifsGenome to find motifs in the hg38 or mm10 genome. Peak set gene annotation was carried out using GREAT (McLean et al., 2010, version 4.0.4) with default parameters. The Enrichr web server (Kuleshov et al., 2016) was used for gene ontology, the Appyter option used to create the volcano plot for enriched GO biological processes using default settings.

## Supplementary Material

10.1242/develop.201124_sup1 Supplementary information
