# Supplementary material for: A new CUT&RUN low volume-urea (LoV-U) protocol optimized for transcriptional co-factors uncovers Wnt/β-catenin tissue-specific genomic targets
Source: Development. 2022 Nov 30;149(23):dev201124. doi: 10.1242/dev.201124 (PMC10112916; doi:10.1242/dev.201124)
Supplement: Supplementary file 1 [file develop-149-201124_review_history.pdf]

A new CUT&RUN low volume-urea (LoV-U) protocol optimized for transcriptional co-factors uncovers Wnt/β-catenin tissue-specific genomic targets

Gianluca Zambanini, Anna Nordin, Mattias Jonasson, Pierfrancesco Pagella and Claudio Cantù
DOI: 10.1242/dev.201124

Editor: Haruhiko Koseki

Review timeline

Original submission:	11 July 2022
Editorial decision:	19 August 2022
First revision received:	17 October 2022
Editorial decision:	26 October 2022
Second revision received:	26 October 2022
Accepted:	26 October 2022

Original submission

First decision letter

MS ID#: DEVELOP/2022/201124

MS TITLE: A New CUT&RUN Low Volume-Urea (LoV-U) protocol uncovers Wnt/&[beta]-catenin tissue-specific genomic targets

AUTHORS: Gianluca Zambanini, Anna Nordin, Mattias Jonasson, Pierfrancesco Pagella, and Claudio Cantu

I have now received all the referees' reports on the above manuscript, and have reached a decision. The referees' comments are appended below, or you can access them online: please go to BenchPress and click on the 'Manuscripts with Decisions' queue in the Author Area.

As you will see, the referees express considerable interest in your work, but have some significant criticisms and recommend a substantial revision of your manuscript before we can consider publication. If you are able to revise the manuscript along the lines suggested, which may involve further experiments, I will be happy receive a revised version of the manuscript. Your revised paper will be re-reviewed by one or more of the original referees, and acceptance of your manuscript will depend on your addressing satisfactorily the reviewers' major concerns. Please also note that Development will normally permit only one round of major revision. If it would be helpful, you are welcome to contact us to discuss your revision in greater detail. Please send us a point-by-point response indicating your plans for addressing the referee's comments, and we will look over this and provide further guidance.

Please attend to all of the reviewers' comments and ensure that you clearly highlight all changes made in the revised manuscript. Please avoid using 'Tracked changes' in Word files as these are lost in PDF conversion. I should be grateful if you would also provide a point-by-point response detailing how you have dealt with the points raised by the reviewers in the 'Response to Reviewers' box. If you do not agree with any of their criticisms or suggestions please explain clearly why this is so.

© 2022. Published by The Company of Biologists under the terms of the Creative Commons Attribution License (https://creativecommons.org/licenses/by/4.0/).

Reviewer 1

Advance summary and potential significance to field

The study describes a simple modification to a popular method for chromatin profiling that has potentially extended its utility to non-DNA-binding protein complexes.

Comments for the author

The authors describe a simple modification of CUT&RUN, which uses an antibody-tethered nuclease for genome-wide chromatin profiling, and is increasing in popularity. This modification was intended to overcome difficulties that the authors encountered using the standard CUT&RUN protocol to profile beta-catenin. It was hypothesized that the reason for the failure with this target was that beta-catenin doesn't bind DNA like transcription factors, nor epitopes on nucleosomes, but rather is part of a large multi-protein complex, and this would hinder release of MNase-digested fragments into the supernatant. Their solution was to follow the CUT&RUN protocol using nuclei, remove the first supernatant, add an 8.8M urea-containing buffer to disrupt proteinaceous complexes to release the fragments targeted by beta-catenin then optionally pool the two fractions and do a bead-based clean-up. While this sounds plausible, there is another explanation for the failure that authors acknowledge, which is that upon activation, beta-catenin builds up in the cytoplasm and that could have short-circuited the antibody and Protein AG-Tn5, which is why the authors used nuclei rather than whole cells. Since the authors did not test standard CUT&RUN with nuclei, but rather used a whole-cell permeabilization protocol, it seems not unlikely that the standard protocol using nuclei would have done just as well. Therefore, it is not clear that adding an 8.8M urea wash rather than the standard CUT&RUN RNase A-containing stop buffer was the key to success. This needs to be tested by using nuclei for CUT&RUN, something that was done for mammalian cells in the original CUT&RUN protocol (eLife, 2017) cited by the authors.

A cursory check of the recent literature shows that some authors have been successful using the standard CUT&RUN protocol on non-DNA-binding proteins. For example in PMID: 35551737 the authors used CUT&RUN to profile Inositol polyphosphate multikinase which is present in both the cytoplasm and nucleus and binds to the large multi-protein SWI/SNF complex. As the cytoplasmic abundance of beta-catenin is not likely to be an issue for most other non-DNA-binding proteins that are present on chromatin it will be important for the authors to show that their modified method outperforms the current protocol for target proteins other than beta-catenin. Otherwise, they are adding steps, such as purification of nuclei and extra washes that might be unnecessary.

Other modifications to the protocol, such as bead clean-up and using strip-tubes are worthwhile, but incremental advances. Although I have no criticisms of the identification of beta-catenin targets described in the paper, this advance is also incremental, although it does provide an example that illustrates application of the method. In summary, CUT&RUN-LoV-U requires more evidence that it is a sufficient advance for publication in Development.

Reviewer 2

Advance summary and potential significance to field

This Techniques and Resources manuscript by Claudio Cantù's group makes a likely significant and fairly novel contribution to the approaches used to study the genetic regulation of embryonic development. The specific contribution is an improvement method (improved CUT&RUN) for detecting chromatin and genome association of the beta-catenin, the downstream component of the canonical Wnt signal transduction pathway, enabling a more reliable identification of direct Wnt target genes, while needing much less material than any previous relevant methods.

But as the authors correctly point out this improved method will more widely be beneficial for studying the chromatin and genome association of any other transcriptional co-factor only indirectly binding DNA. The examples presented in the context of Wnt signalling (e.g. comparing beta-catenin with TCF/LEF association with chromatin or genome), provide tantalising insight how

© 2022. Published by The Company of Biologists under the terms of the Creative Commons Attribution License (https://creativecommons.org/licenses/by/4.0/).

this improved method could be applied to uncovering important features of Wnt signalling controlling embryonic development (ie., tissue-specific Wnt target gene regulation).

Comments for the author

There are no concerns of mine that I think couldn't be addressed in revisions to make the manuscript in approximately the current form suitable for the Techniques and Resources section of DEVELOPMENT.

I have a concern with the TCF7L2 data as it is currently shown. The authors are apparently only showing primary evidence of results on TCF7L2 (C&R-LoV-U, Figure 3) that either appear to show absence of overlap with detected beta-catenin "peaks" (Fig.3E, AXIN2, UTP6, TSPAN12) or apparent TCF7L2 "peaks" where there is an absence of any beta-catenin "peak" (Fig.3E, PIGS). From the primary data presented, it is not absolutely clear whether there is an absence of evidence (i.e. the TCF7L2 C&R-LoV-U is maybe not working well, experiment hasn't worked) or actual evidence of absence (i.e. that TCF7L2 doesn't bind where beta-catenin indirectly interacts with genetic loci (and vice versa in Fig.3E, PIGS). There appears to be a positive control missing.

The Venn diagram (part of Fig.3D, far right) does suggest that the authors have evidence that some TCF7L2 and beta-catenin "peaks" overlap. The authors should consider providing primary evidence of an example in Fig.3E of a locus where TCF7L2 and beta-catenin "peaks" overlap. If however, the TCF7L2 C&R-LoV-U was not working well, then the possibly misleading TCF7L2 data could just be removed, without, I think, fundamentally affecting the suitability of the manuscript for publication as a Techniques & Resources article.

Minor suggestions to the authors:
- Consider whether the use of the term "ontogenically" (at the end of abstract and at the end of introduction) is a bit outdated, and whether more contemporary terms such as "embryonic development" would be more familiar to your readers.
- Consider introducing the binding of protein A and protein G to antibodies for those readers not already familiar with Cut&Run (e.g. page 3, line 2).
- Consider explaining "CHIR stimulation" for those readers not already familiar with small molecule drugs for experimentally manipulating Wnt signalling.
- Consider explaining "chaotropic" (e.g. page 4, line 18) to those readers not already familiar with biochemical extraction methods, and also that Urea is such a chaotropic agent (e.g. page 4, line 21).
- In the context of readers possibly wanting to be using your method for identification of direct Wnt target genes, consider referring specifically to "protein targets" (e.g. page 5, line 18) when considering wider application of the improved method (C&R-LoV-U).
- I think you wanted to refer to Figure 3B (not 2B, page 5, line 29).
- When you mention Ets2 and Ezh2 (page 6, line 32), it is not clear which figure you are referring to, should this be Fig.4A?
- When you mention Tle1 (page 7, line 18 onward), it is not clear what point you are making, the fact that a Wnt pathway component is encoded by a direct Wnt target gene suggests a (negative in this case) feedback loop. If that is your point, consider spelling it out.
- In the Figures it is not always clear which panel lettering applies to which panels, I assume Fig.1A shows both AXIN2 and LEF1 data, but maybe a border or sub panel lettering (A1 and A2, or the like) may make it easier to follow. (also Figure 1 C bottom panels?; Figure 3, panels C, D and E; and Figure 5, panels C and D).

Reviewer 3

Advance summary and potential significance to field

This manuscript describes an important modification of the Cut & Run method that has the potential to gain widespread usage among developmental biologists and other researchers interested in gene regulation. Picking beta-catenin to vet this approach is appropriate, while there are reports of high-quality ChIPseq data sets for beta-catenin (including from the senior author) it requires that a high level of technical expertise. As pointed out by the authors, Cut & Run doesn't

© 2022. Published by The Company of Biologists under the terms of the Creative Commons Attribution License (https://creativecommons.org/licenses/by/4.0/).

provide the answer, but their clever modifications, informed by biochemical considerations have provided a new way for researchers to obtain genome-wide surveys for beta-catenin and other proteins.

Comments for the author

I have several comments that I recommend the authors address in a revision:
1) I think the authors overstate the utility of ChiPseq/C &R data sets for identifying direct targets of particular inputs (in this case Wnt signaling).
These data, when done well, provide a description of the physical location of a protein, not it's functional potential. In the case of beta-catenin, the revised manuscript should cite Nakamura et al 2016 & 2017, which showed that only a small faction of beta-catenin ChIPseq peaks in Xenopus embryos appear to be functional (as judged by Wnt-dependence of the expression of nearby genes). Not for inclusion in this paper, but the authors should take a look at the work of Visel and Pennacchio with p300, where they found the majority of ChIPseq peaks were at locations with enhancer activity in mouse embryos (Visel et al 2009; Blow et al 2010). Some language to indicate that a genome-wide survey of protein localization on chromatin, while impressive and incredibly useful, is not a stand alone indicator of the presence of a regulated enhancer.

2) I appreciate the comparison between the published beta-catenin ChIPseq data in Doumpas et al 2019 and the new C&R-LoV-U data, but would also like to see a more holistic comparision. How many of the high confidence ChiPseq peaks were identified with the new method, and how many of the high confidence C&R-LoV-U peaks were identified in Doumpas et al.?

3) It would be nice to know the beta-catenin peaks in HEK293T cells not treated with CHIR. It would be another control to confirm the specificity of the identified binding regions.

4) The authors express surprise about the lack of overlap between beta-catenin binding regions in HEK293T and murine hindlimb. While I don't have a single reference to support this, I believe there is ample evidence that TFs and coactivator distributions in genomes are highly cell-type specific. Having written that, I appreciate the authors focused examination of some Wnt targets in hindlimbs, as a cautionary tale for solely relying on statistical calling methods (though they are less biased).

5) The genome snap shots in the hindlimb (Fig. 4) are less clean than those in HEK293T cells. This is no surprise given the cell-type heterogeneity of the latter, but can the authors comment on this in the results/discussion section of the revised manuscript?

6) In the legend for Figure 3, there is no "C" indicated.

7) In Figure 4C, there appears to be a mistake in labeling the enriched motifs: the fourth sequence logo shown clearly fits the TCF binding site consensus, while the second one does not.

First revision

Author response to reviewers' comments

DETAILED RESPONSE TO THE REVIEWERS
We are grateful to the referees for their detailed evaluation of our data and the resulting conclusions. Integrating their comments helped us to significantly improve the strength of our protocol and findings. Below we describe how we have addressed each of the points that were raised. The referees' comments are displayed in numerical order, and our response to each of them is marked by "RESPONSE". To allow easier tracking of the changes implemented, we have also marked in red the parts that we have modified or added in the manuscript.

© 2022. Published by The Company of Biologists under the terms of the Creative Commons Attribution License (https://creativecommons.org/licenses/by/4.0/).

Response to Reviewer #1:

1) The authors describe a simple modification of CUT&RUN, which uses an antibody-tethered nuclease for genome-wide chromatin profiling, and is increasing in popularity. This modification was intended to overcome difficulties that the authors encountered using the standard CUT&RUN protocol to profile beta-catenin. It was hypothesized that the reason for the failure with this target was that beta-catenin doesn't bind DNA like transcription factors, nor epitopes on nucleosomes, but rather is part of a large multi-protein complex, and this would hinder release of MNase-digested fragments into the supernatant. Their solution was to follow the CUT&RUN protocol using nuclei, remove the first supernatant, add an 8.8M urea-containing buffer to disrupt proteinaceous complexes to release the fragments targeted by beta-catenin, then optionally pool the two fractions and do a bead-based clean-up.
RESPONSE – We are grateful to this referee for the careful analysis of our protocol. As outlined below, we have strived to address each of the criticisms and comments raised.
While this sounds plausible, there is, another explanation for the failure that authors acknowledge, which is that upon activation, beta-catenin builds up in the cytoplasm and that could have short-circuited the antibody and Protein AG-Tn5, which is why the authors used nuclei rather than whole cells. Since the authors did not test standard CUT&RUN with nuclei, but rather used a whole-cell permeabilization protocol, it seems not unlikely that the standard protocol using nuclei would have done just as well. Therefore, it is not clear that adding an 8.8M urea wash rather than the standard CUT&RUN RNase A-containing stop buffer was the key to success. This needs to be tested by using nuclei for CUT&RUN, something that was done for mammalian cells in the original CUT&RUN protocol (eLife, 2017) cited by the authors.

RESPONSE – The point raised here is of extreme importance, and we now realize having previously failed to clearly mention it in detail in our manuscript. Indeed, several of our initial attempts in using CUT&RUN to profile ß-catenin were de facto performed on ConcanavalinA-immobilized nuclei as suggested in the original protocol (Skene and Henikoff, eLife, 2017 - that is, not only using digitonin-permeabilized cells as in Skene et al., Nat. Protocols, 2018). However, no reliable ß-catenin profile had been achieved. This was precisely what generated the initial impetus of designing novel strategies that ultimately brought us to develop the LoV-U protocol.
As we recognize the centrality of this concern, and the importance of clarifying it experimentally, during the revision time we have performed additional experiments such that we could directly compare the three CUT&RUN variants: the 2017 protocol (nuclear extraction), the 2018 version (whole cells permeabilized with digitonin) and LoV-U. We have added this new comparative dataset in the revised Figure 1A: whilst with the 2018 (digitonin) protocol we systematically failed in producing a ß-catenin track, the 2017 protocol, likely thanks to nuclear extraction as the reviewer suggests, yielded a few instances of correctly located yet low-intensity signal. This, however, was robustly outperformed by the LoV-U protocol, which allowed us to detect a broad set of high-signal peak regions.
It is worth pointing out that the original protocol has been used by Skene and Henikoff (eLife, 2017) to profile chromatin associated complexes. However, to this aim, the authors described “(we) found it necessary to extract total DNA rather than chromatin solubilized by CUT&RUN in situ, which may be too large to diffuse through nuclear pores. Therefore, we extracted all DNA and preferentially removed large DNA fragments with AMPure beads”.
Our interpretation is that the in situ protein denaturation via urea that we introduce here exerts an analogous effect but does not require long procedures that include whole genome purification and subsequent costly bead-mediated short fragment enrichment. We have added a short explicative sentence about this interpretation in the revised version of our manuscript (page 5, lines 2-7).

2) A cursory check of the recent literature shows that some authors have been successful using the standard CUT&RUN protocol on non-DNA-binding proteins. For example in PMID: 35551737 the authors used CUT&RUN to profile Inositol polyphosphate multikinase which is present in both the cytoplasm and nucleus and binds to the large multi-protein SWI/SNF complex. As the cytoplasmic abundance of beta-catenin is not likely to be an issue for most other non-DNA-binding proteins that are present on chromatin, it will be important for the authors to show that their modified method outperforms the current protocol for target proteins other than beta-catenin. Otherwise, they are adding steps, such as purification of nuclei and extra washes that might be unnecessary.

© 2022. Published by The Company of Biologists under the terms of the Creative Commons Attribution License (https://creativecommons.org/licenses/by/4.0/).

RESPONSE – This valuable suggestion prompted us to compare the two protocols on additional, non-DNA-binding targets. We selected CBP (CREB binding protein) and HDAC1, both co-factors that are known to be relevant to Wnt target genes activation and repression, respectively (revised Figure 3C). We confirm that original CUT&RUN could reveal reliable genome-wide tracks. However, when comparing the signal intensity in CUT&RUN versus C&R-LoV-U, we were surprised in noticing that not only C&R-LoV-U could recapitulate the original CUT&RUN results, but that it discovers an additional set of binding regions that CUT&RUN did not detect. Most notably, this fraction of peaks represents the group displaying the highest signal-to-noise ratio, revealing that urea administration is well-suited in extracting DNA fragments from those chromatin regions that are accessible to pAG-MNase but fully inhibits release of the cut DNA molecules. We speculate that this might underlie a biologically interesting aspect concerning the co-factors genome-wide distribution: these proteins could be recruited at genomic loci having different biophysical properties (phase-separated) and/or more simply a higher protein concentration. It is plausible that the set of targets identified with C&R-LoV-U, but not with classical CUT&RUN, is characterized by local crowding or by the presence of protein complexes of high molecular mass. We have added this interpretation at page 6 lines 11-17 of the revised manuscript.
Overall, we are grateful for this suggestion, as the new experiments reveal the importance of this technology improvement and provide compelling evidence that C&R-LoV-U protocol might be preferable to classical CUT&RUN when applied to non-DNA-binding transcriptional co-factors. Based on these new evidence, we considered appropriate to emphasize this aspect in the title, where we have added the specification that this protocol is generally optimized for non-DNA-binding transcriptional co-factors.
Finally, we considered fair to acknowledge the article mentioned, where the authors profile the non-DNA-binding IPMK (PMID: 35551737), and included it in our reference list. However, inspection of their datasets revealed an overall low signal-to-noise ratio even in those regions that have been selected for representation (PMID: 35551737/Beon et al., 2022: look at their Figure 4—figure supplement 2E and Figure 5—figure supplement 1B). Among all the datasets presented there, the IPMK profile is performed as single replicate and, overall, is the one with the least persuasive signal. This, in our view, reflects the general difficulty to reliably detect this class of proteins by standard protocols, and emphasizes the need of method development that provided the impetus to fine-tune LoV-U.

3) Other modifications to the protocol, such as bead clean-up and using strip-tubes are worthwhile, but incremental advances. Although I have no criticisms of the identification of beta-catenin targets described in the paper, this advance is also incremental, although it does provide an example that illustrates application of the method. In summary, CUT&RUN-LoV-U requires more evidence that it is a sufficient advance for publication in Development

RESPONSE – We agree with the reviewer that the additional modifications (smaller volumes, gel-DNA extraction and beads clean-up) constitute minor advances. However, we are keen in describing them in detail, as they might facilitate other researchers in performing and scaling up their experimental plans without the need of expensive – and often non-affordable – pipetting robots. We believe and hope that, thanks to the new data presented in response to this referee's points 1 and 2, namely by showing that i) urea administration provides a key advantage, and that ii) there is a critical improvement in the detection efficiency of new peaks when targeting transcriptional co-factors, both quantitatively and qualitatively, several new researchers in several fields will be able to generate reliable genome-wide binding tracks of co-factors more easily and reproducibly. And advance that we deem considerable for publication in a high-quality and broad readership journal.

Response to Reviewer #2:

This Techniques and Resources manuscript by Claudio Cantù's group makes a likely significant and fairly novel contribution to the approaches used to study the genetic regulation of embryonic development. The specific contribution is an improvement method (improved CUT&RUN) for detecting chromatin and genome association of the beta-catenin, the downstream component of the canonical Wnt signal transduction pathway, enabling a more reliable identification of direct Wnt target genes, while needing much less material than any previous relevant methods. But as the authors correctly point out this improved method will more widely be beneficial for studying the chromatin and genome association of any other transcriptional co-factor only indirectly binding

© 2022. Published by The Company of Biologists under the terms of the Creative Commons Attribution License (https://creativecommons.org/licenses/by/4.0/).

DNA. The examples presented in the context of Wnt signalling (e.g. comparing beta-catenin with TCF/LEF association with chromatin or genome), provide tantalising insight how this improved method could be applied to uncovering important features of Wnt signalling controlling embryonic development (ie., tissue-specific Wnt target gene regulation).

RESPONSE – We are grateful to this reviewer for the careful summary of the scientific rationale of our paper and for the positive take on the reliability and the potential future uses of the new protocol. As detailed below, we did our best to address each point raised and firmly believe that, in doing so, we have now produced a stronger, more convincing manuscript.
1) I have a concern with the TCF7L2 data as it is currently shown. The authors are apparently only showing primary evidence of results on TCF7L2 (C&R-LoV-U, Figure 3) that either appear to show absence of overlap with detected beta-catenin "peaks" (Fig.3E, AXIN2, UTP6, TSPAN12) or apparent TCF7L2 "peaks" where there is an absence of any beta-catenin "peak" (Fig.3E, PIGS). From the primary data presented, it is not absolutely clear whether there is an absence of evidence (i.e. the TCF7L2 C&R-LoV-U is maybe not working well, experiment hasn't worked) or actual evidence of absence (i.e. that TCF7L2 doesn't bind where beta-catenin indirectly interacts with genetic loci (and vice versa in Fig.3E, PIGS). There appears to be a positive control missing. The Venn diagram (part of Fig.3D, far right) does suggest that the authors have evidence that some TCF7L2 and beta-catenin "peaks" overlap. The authors should consider providing primary evidence of an example in Fig.3E of a locus where TCF7L2 and beta-catenin "peaks" overlap. If however, the TCF7L2 C&R-LoV-U was not working well, then the possibly misleading TCF7L2 data could just be removed, without, I think, fundamentally affecting the suitability of the manuscript for publication as a Techniques & Resources article.

RESPONSE – The reviewer is correct in pointing out that our overall analysis indicates that TCF7L2 gave us reliable tracks, and it is important to show some instance of this. We now display this as part of the main figures (Figure 3F): one example of a locus (NFIA) in which TCF7L2 signal is detectable and is accompanied by simultaneous (that is, in the same position) of ß-catenin. While, based on our data, TCF7L2 could appear as being less frequently associated to classical Wnt targe regions, a plausible phenomenon consistent with the previously recognized tissue-specific participation in the Wnt signalling cascade of the four TCF/LEF (PMID: 17143293; PMCID: PMC7275771), we also wish to point out that the different antibodies that we use for each of these factors possess apparent different efficiencies or specificity for their epitope. Hence, in the absence of new evidence, which we are trying to gather but that go beyond the scope of the current study, we prefer to be cautious and not draw any particularly strong conclusion based on this.

Minor comments

2) Consider whether the use of the term "ontogenically" (at the end of abstract and at the end of introduction) is a bit outdated, and whether more contemporary terms such as "embryonic development" would be more familiar to your readers.

RESPONSE – We agree that this is a good way to facilitate reading, and we have strived to improve on all the suggestions proposed by this referee in the revised version of our manuscript (see below). However, in this case, we are keen in using "ontogenetically": despite being a quite rarely used term, we feel that it encloses a broader spectrum of processes than "embryonic development", among which morphogenesis, the establishment of tissue-homeostasis, regeneration, aging and any other process that occur throughout the lifetime of an organisms. We hope that our CUT&RUN-LoV-U will be applied in such broad spectrum of fields!

3) Consider introducing the binding of protein A and protein G to antibodies for those readers not already familiar with Cut&Run (e.g. page 3, line 2).

RESPONSE – We agree with implementing this explanation. In the revised manuscript we now introduce this better by saying (revised page 3): "C&R relies on the antibody-mediated recognition of specific target factor by the fusion of proteinA/G, a recombinant protein combining the affinity for the heavy antibody chains of both Protein A and Protein G, with micrococcal nuclease (pAG-MN), which cuts the DNA in a sequence-independent manner upon addition of Ca2+. When activated, pAG-MN cleaves the DNA underlying target TFs and generates short fragments that that

© 2022. Published by The Company of Biologists under the terms of the Creative Commons Attribution License (https://creativecommons.org/licenses/by/4.0/).

diffuse in the supernatant, can be harvested and sequenced, before mapping onto a reference genome – producing TF-specific genome-wide binding patterns (Meers et al., 2019a).”

4) Consider explaining "CHIR stimulation" for those readers not already familiar with small molecule drugs for experimentally manipulating Wnt signalling.

RESPONSE – Based on this suggestion, we have inserted an introductory sentence that clarifies the action of CHIR by saying, at the beginning of the results: “A reliable method to trigger Wnt/ß-catenin activation is treatment with CHIR99021 (referred to as CHIR), a potent GSK3 inhibitor that causes ß-catenin stabilization”

5) Consider explaining "chaotropic" (e.g. page 4, line 18) to those readers not already familiar with biochemical extraction methods, and also that Urea is such a chaotropic agent (e.g. page 4, line 21).

RESPONSE – We now briefly explain what chaotropic agents do by saying (page 4, line 21-22): “On the other hand, while nuclei isolation alone only marginally improved the final yield (Fig. 1A), it was the subsequent implementation of chaotropic agents – molecules that cause protein denaturation – that when applied after pAG-MN cleavage enabled the release and harvest of a considerably higher amount of DNA fragments that correctly mapped to WNT Reponsive Elements (WRE) in the vicinity of WNT target genes”.

6) In the context of readers possibly wanting to be using your method for identification of direct Wnt target genes, consider referring specifically to "protein targets" (e.g. page 5, line 18) when considering wider application of the improved method (C&R-LoV-U).

RESPONSE – We consider this an important specification to avoid misunderstanding. We have now added “protein” when referring to our experimental target – as opposed to target genes identified in CUT&RUN experiments.

7) I think you wanted to refer to Figure 3B (not 2B, page 5, line 29).

RESPONSE – We acknowledge the mistake, and we corrected the reference, changing it from 2B to 3B.

8) When you mention Ets2 and Ezh2 (page 6, line 32), it is not clear which figure you are referring to, should this be Fig.4A?

RESPONSE – We now refer to Fig. 4A when mentioning the tracks relative of these two genes.

9) When you mention Tle1 (page 7, line 18 onward), it is not clear what point you are making, the fact that a Wnt pathway component is encoded by a direct Wnt target gene suggests a (negative in this case) feedback loop. If that is your point, consider spelling it out.

RESPONSE – We now spell out this more clearly by writing: ”Among these: Tle1, encoding for the Transducin-Like Enhancer Of Split/Groucho repressor is known to bind on TCF/LEF at WREs and repress transcription of WNT target genes, pointing to a potential novel negative-feedback mechanism”.

10) In the Figures it is not always clear which panel lettering applies to which panels, I assume Fig.1A shows both AXIN2 and LEF1 data, but maybe a border or sub panel lettering (A1 and A2, or the like) may make it easier to follow. (also Figure 1 C bottom panels?; Figure 3, panels C, D and E; and Figure 5, panels C and D).

RESPONSE – We have improved our figure representation, and we believe that each letter is now unambiguously associated to a specific panel or set of panels.

Response to Reviewer #3:

© 2022. Published by The Company of Biologists under the terms of the Creative Commons Attribution License (https://creativecommons.org/licenses/by/4.0/).

This manuscript describes an important modification of the Cut & Run method that has the potential to gain widespread usage among developmental biologists and other researchers interested in gene regulation. Picking beta-catenin to vet this approach is appropriate, while there are reports of high-quality ChIPseq data sets for beta-catenin (including from the senior author) it requires that a high level of technical expertise. As pointed out by the authors, Cut & Run doesn't provide the answer, but their clever modifications, informed by biochemical considerations have provided a new way for researchers to obtain genome-wide surveys for beta-catenin and other proteins.
RESPONSE – We appreciate the reviewer for this accurate summary of our study, and for emphasizing the relevance of our new protocol. This reviewer raises important points which we have strived to address adequately, as outlined below.

1) I think the authors overstate the utility of ChiPseq/C&R data sets for identifying direct targets of particular inputs (in this case Wnt signaling). These data, when done well, provide a description of the physical location of a protein, not it's functional potential. In the case of beta-catenin, the revised manuscript should cite Nakamura et al 2016 & 2017, which showed that only a small faction of beta-catenin ChIPseq peaks in Xenopus embryos appear to be functional (as judged by Wnt-dependence of the expression of nearby genes). Not for inclusion in this paper, but the authors should take a look at the work of Visel and Pennacchio with p300, where they found the majority of ChIPseq peaks were at locations with enhancer activity in mouse embryos (Visel et al 2009; Blow et al 2010). Some language to indicate that a genome-wide survey of protein localization on chromatin, while impressive and incredibly useful, is not a stand alone indicator of the presence of a regulated enhancer.

RESPONSE – This is an important specification that we now desire to indicate as limitation of our study. More precisely, after having discussed the newly discovered tissue-specific target genes at the end of our results and discussion section, we write: "We wish to point out that the identification of tissue-specific physical targets of ß-catenin does not imply that these constitute functionally relevant transcribed genes, as previous studies have shown that context-dependent mechanisms are necessary for transcription after ß-catenin recruitment (Nakamura and Hoppler, 2017; Nakamura et al., 2016)"

2) I appreciate the comparison between the published beta-catenin ChIPseq data in Doumpas et al 2019 and the new C&R-LoV-U data, but would also like to see a more holistic comparision. How many of the high confidence ChiPseq peaks were identified with the new method, and how many of the high confidence C&R-LoV-U peaks were identified in Doumpas et al.?

RESPONSE – A more careful comparison between our CUT&RUN-LoV-U high-confidence targets with the peaks identified in Doumpas at al. yielded an overlap of 243 common target genes (accompanied by 942 unique ChIP-seq genes and 331 unique CUT&RUN hits). The overlap set, when analyzed with gene ontology, displays an increase enrichment of terms related to Wnt signaling pathway than in the two individual datasets, suggesting that merging the results of these two technologies might lead to an even more robust set of consistent direct targets.
We decided however not to include this analysis in the final version of the manuscript. A thorough comparison between ChIP-seq and CUT&RUN has already been proposed in the original paper (Skene and Henikoff, 2017), and it is not the focus of our study. Moreover, ChIP-seq and CUT&RUN are executed via different biochemistries, and a low overlap between peaks, their exact positions and sizes, could be explained by several factors (a point that has been fueling long discussion in our laboratory). For example cross-linking, in ChIP-seq, presumably increases the number of protein-DNA interactions between the target and the DNA regulatory regions that become proximal during gene regulation and are therefore cross-linked via formaldehyde to the directly-bound target site, generating a higher number of peaks. Moreover, sonication and subsequent whole-genome DNA purification typical of ChIP-seq produces a different pattern of DNA fragments than the MNase-mediated cuts that occurs only from specific positions in CUT&RUN. These and many other factors heavily affect computational peak calling. It is from this that stemmed the necessity to develop SEACR, a novel peak caller for CUT&RUN (Meers et al., 2019).

3) It would be nice to know the beta-catenin peaks in HEK293T cells not treated with CHIR. It would be another control to confirm the specificity of the identified binding regions.

© 2022. Published by The Company of Biologists under the terms of the Creative Commons Attribution License (https://creativecommons.org/licenses/by/4.0/).

RESPONSE – We agree that this is an even better control than the IgG track previously added. We have performed this experiment in the context of another project and have now included it in the current manuscript as new track displaying β-catenin distribution in HEK293T treated with LGK (a drug that inhibits the secretion of all WNT proteins), and in the absence of CHIR-mediated stimulation (revised Fig 1B). Moreover, we have used this dataset to computationally subtract further additional potential false positive peaks from all the other tracks. This yielded an even "cleaner" set of target regions, which we used for all the subsequent analyses.

4) The authors express surprise about the lack of overlap between beta-catenin binding regions in HEK293T and murine hindlimb. While I don't have a single reference to support this, I believe there is ample evidence that TFs and coactivator distributions in genomes are highly cell-type specific. Having written that, I appreciate the authors focused examination of some Wnt targets in hindlimbs, as a cautionary tale for solely relying on statistical calling methods (though they are less biased).

RESPONSE – The referee precisely refers an aspect central of our research, to which our laboratory is dedicating intense efforts. See, for example, our recent review article whose aim is to precisely discuss this problem (Söderholm and Cantù, 2020). We agree that there is a considerable body of evidence showing that TFs, together with co-factors, are involved in a combinatorial control of gene expression. However, this important feature of transcriptional control has been often overlooked in the field of signalling cascades, in favor of a more conservative view in which these are executed as a linear series of events. This conservative model would predict a large – if not complete – overlap of target genes across tissues. This is contrary to our finding, and we consider important to emphasize it.
In addition, we did expect to identify "some" tissue-specific target loci; our surprise was caused by the extent of this phenomenon – namely that the vast majority of targets are not shared across the models that we tested. We feel that our description of this in the manuscript, faithfully reflects this view.

5) The genome snap shots in the hindlimb (Fig. 4) are less clean than those in HEK293T cells. This is no surprise given the cell-type heterogeneity of the latter, but can the authors comment on this in the results/discussion section of the revised manuscript?

RESPONSE – We agree with the relevance of this specification. In the revised manuscript, we have now edited a description of target genes in hindlimbs as follows (lines 19-24, page 7): "..by using the same statistical parameters, Axin2 was not called as a gene-associate peak in the hindlimb despite the consistent signal enrichment observed at its promoter (Fig. 4B). This is possibly caused by the intrinsically lower signal-to-noise ratio obtained when targeting β-catenin in mouse hindlimb cells, likely reflecting the higher heterogeneity of this cell population in comparison to HEK293T, and the small proportion of cells with physiologically active WNT/β-catenin signalling (Maretto et al., 2003)"

6) In the legend for Figure 3, there is no "C" indicated.

RESPONSE – We have corrected this, thanks!

7) In Figure 4C, there appears to be a mistake in labeling the enriched motifs: the fourth sequence logo shown clearly fits the TCF binding site consensus, while the second one does not.

RESPONSE – Thanks for the attentive recognition of the position weight matrices. We have corrected the mistake and substituted the fourth consensus sequence with the appropriate FOXA2 one resulting from HOMER.

© 2022. Published by The Company of Biologists under the terms of the Creative Commons Attribution License (https://creativecommons.org/licenses/by/4.0/).

Second decision letter

MS ID#: DEVELOP/2022/201124

MS TITLE: A New CUT&RUN Low Volume-Urea (LoV-U) protocol optimized for transcriptional co-factors uncovers Wnt/&[beta]-catenin tissue-specific genomic targets

AUTHORS: Gianluca Zambanini, Anna Nordin, Mattias Jonasson, Pierfrancesco Pagella, and Claudio Cantu

I have now received all the referees reports on the above manuscript, and have reached a decision. The referees' comments are appended below, or you can access them online: please go to BenchPress and click on the 'Manuscripts with Decisions' queue in the Author Area.

The overall evaluation is positive and we would like to publish a revised manuscript in Development, provided that referee 1's comments can be addressed. Please attend to all of the reviewers' comments in your revised manuscript and detail them in your point-by-point response. If you do not agree with any of their criticisms or suggestions explain clearly why this is so. If it would be helpful, you are welcome to contact us to discuss your revision in greater detail. Please send us a point-by-point response indicating your plans for addressing the referee's comments, and we will look over this and provide further guidance.

Reviewer 1

Advance summary and potential significance to field

CUT&RUN is becoming increasingly popular and the authors have demonstrated that with their simple modification of the protocol they can recover target sites of transcriptional co-factors that were previously intractable.

Comments for the author

I am satisfied with authors' responses and revisions. Showing that their modification of CUT&RUN released CBP and HDAC1 fragments that had the highest signal-to-noise is persuasive (and interesting) evidence that their modification of the method was required to release fragments from especially crowded regions of the genome.

Figure 3C: Headings for the signal enrichment plots are missing in the first panel. Also, they appear here and elsewhere to have been clustered (k-means perhaps?), and if so, the number of clusters should be specified in the legend and more detail is needed in the Data Analysis section.

Reviewer 3

Advance summary and potential significance to field

The authors make significant modifications to the standard Cut and Run protocol to significantly increase it utility for non-DNA binding proteins. Even if limited to beta-catenin (which is doubtful) I think this is a significant advance.

Comments for the author

I think the authors have adequately addressed my concerns and I recommend that the revised manuscript be accepted.

© 2022. Published by The Company of Biologists under the terms of the Creative Commons Attribution License (https://creativecommons.org/licenses/by/4.0/).

Second revision

Author response to reviewers' comments

We are grateful for the careful analysis on our revised manuscript, and we are glad to see that the additional evidence presented persuasively displayed the improvements provided by our protocol.

Reviewer 3 commented: "Figure 3C: Headings for the signal enrichment plots are missing in the first panel. Also, they appear here and elsewhere to have been clustered (k-means perhaps?), and if so, the number of clusters should be specified in the legend and more detail is needed in the Data Analysis section."
We have now corrected the figure panel 3C, by adding the headings to each heatmap. Moreover, we have specified how the signal enrichment entries are ranked, by writing "Signal entries in the heatmap are ordered by overall enrichment of the first profile."
In order to generate these heatmap charts, we have used default settings, which are now specified in the materials and methods section.

We are grateful for this additional comment, which allowed us to correct an imprecision present in the previous version of the manuscript and figure 3C.

Third decision letter

MS ID#: DEVELOP/2022/201124

MS TITLE: A New CUT&RUN Low Volume-Urea (LoV-U) protocol optimized for transcriptional co-factors uncovers Wnt/β-catenin tissue-specific genomic targets

AUTHORS: Gianluca Zambanini, Anna Nordin, Mattias Jonasson, Pierfrancesco Pagella, and Claudio Cantu

I am happy to tell you that your manuscript has been accepted for publication in Development, pending our standard ethics checks.

© 2022. Published by The Company of Biologists under the terms of the Creative Commons Attribution License (https://creativecommons.org/licenses/by/4.0/).
